# Medical Convention at Rockford, Ill.

**Published:** 1846-06

**Authors:** 


ARTICLE XIV.

**MEDICAL CONVENTION AT ROCKFORD, ILL.**

The adjourned meeting of the "Rock River Medical Society" took place according to notice, on Tuesday the 19th inst., at Rockford. We were present and were highly gratified by the zeal and unanimity exhibited, and the numerous attendance. It convinced us more than ever of the utility of meetings among physicians.

The address of the President, Dr. Goodhue, exhibited many interesting facts in regard to the early history of medicine in Northern Illinois, and contained sound and liberal views in reference to the conduct of practitioners. We hope hereafter to be permitted to lay some of those before our readers.

As this is the first Medical Society organized upon so extensive a scale in this region, we shall publish at length the constitution and laws in our next—they not having come to hand in season for the present No. D. B.

